# A one health approach to tackling AMR and why gender matters: findings from pastoralist communities in Tanzania

**DOI:** 10.3389/fgwh.2024.1429203

**Published:** 2024-07-18

**Authors:** Violet Barasa

**Affiliations:** Institute of Development Studies, University of Sussex, Brighton, United Kingdom

**Keywords:** gender, AMR, one health, LMICs, pastoralism, Tanzania, antibiotics, social drivers

## Abstract

**Introduction:**

Inappropriate use of antimicrobials is a major driver of AMR in low-resource settings, where the regulation of supply for pharmaceuticals is limited. In pastoralist settings in Tanzania, men and women face varying degrees of exposure to antibiotics due to gender relations that shape access and use of antimicrobials. For example, critical limitations in healthcare systems in these settings, including inadequate coverage of health services put people at risk of AMR, as families routinely administer self-treatment at home with antimicrobials. However, approaches to understanding AMR drivers and risk distribution, including the One Health approach, have paid little attention to these gender considerations. Understanding differences in access and use of antimicrobials can inform interventions to reduce AMR risk in community settings. This paper focuses on the gendered risk of AMR through a study of gender and social determinants of access to and use of antimicrobials in low-resource pastoralist settings in Tanzania.

**Methods:**

A mixed methods approach involving household surveys, interviews and ethnographic participant observation in homes and sites of healthcare provision was used, to investigate access and administration of antibiotics in 379 adults in Naiti, Monduli district in northern Tanzania. A purposive sampling technique was used to recruit study participants and all data was disaggregated by sex, age and gender.

**Results:**

Gender and age are significantly associated with the use of antibiotics without a prescription in the study population. Young people aged 18-24 are more likely to use unprescribed antibiotics than older people and may be at a higher risk of AMR. Meanwhile, although more men purchase unprescribed antibiotics than women, the administration of these drugs is more common among women. This is because men control how women use drugs at the household level.

**Discussion:**

AMR interventions must consider the critical importance of adopting and implementing a gender-sensitive One Health approach, as gender interacts with other social determinants of health to shape AMR risk through access to and use of antimicrobials, particularly in resource-limited pastoralist settings.

## 1 Introduction

This paper focuses on the gendered risk of AMR in community settings, through a study of gender and social determinants of access to and use of antimicrobials in low-resource pastoralist settings in Tanzania. It is based on an ethnographic and mixed-methods study of access and use of antibiotics in 379 pastoralists in Naiti, Monduli district, between 2016 and 2017. The study finds that gender and age are significantly associated with the use of antibiotics without a prescription. Younger people aged 18–24 (both male and female) are more likely to use unprescribed antibiotics than older people. The paper proposes that gender needs to be integrated into One Health approaches to understand and address the risk of AMR in pastoralist settings.

## 2 Background: AMR and one heath in community settings

Antimicrobial resistance, the process by which microbes change to survive the drugs designed to treat them, remains a global health challenge for humans, animals and the environment. AMR was associated with an estimated five million deaths in 2019, and if left unchecked, it could have a catastrophic impact on people and the economy (1). The United Nations Environment Programme (UNEP) warns that AMR could shave US$ 3.4 trillion off global GDP annually, and push 24 million people into extreme poverty, due to productivity losses and increased healthcare costs in the next decade (2). Thus, AMR risks eroding our ability to tackle a wide range of infectious diseases including pneumonia, tuberculosis, and malaria.

In many low-income communities in sub-Saharan Africa, particularly in remote, medically underserved pastoralist communities, an intersection of poverty and its determinants (poor sanitation, lack of clean water and hygiene) can increase the risks of developing AMR, due to habitat overlaps between people, livestock and the environment, which enhance disease spillovers from both wild and domestic animals to humans, and delayed and/or inappropriate treatment can lead to the development of AMR (3, 4). Human exposure to AMR in the environment can also occur through contact with polluted waters, contaminated food, inhalation of fungal spores, and other pathways that contain antimicrobial-resistant microorganisms (2).

However, for resource-poor and marginalised communities in many LMICs, it is poverty and socio-political (structural) factors that feed the “silent” epidemic that is AMR. In these contexts, research on the real scale of the problem or its impacts is missing due to disinterest from funders and policymakers. The example of pastoralists living in remote, hard-to-reach settings in northern Tanzania, where healthcare infrastructure is non-existent, brings home these structural determinants of AMR. Unregulated vendors supply antimicrobials to families without access to alternative or safer healthcare options (5, 6). AMR is thus an equity issue, as it disproportionately impacts Low-Income Countries, particularly populations facing structural marginalisations (7, 8).

Farmer [(8): 1] discusses how poverty and its conditions- poor housing, inaccessible healthcare, and economic and political marginalisation to name but a few- conjure up to shape the course of illness and health in both individuals and populations. He described this as “structural violence”, defining it as social arrangements that put individuals and populations in harm's way. They are “structural” because they are embedded in the political and social organisation of the social worlds of people in particular places and not others. They are “violent” because they cause harm (illness, disability and even death) to the people who are usually already on the margins of society- women, children, people living with disabilities and or co-comorbidities, and the elderly).

AMR thus presents a perfect example in which the structural factors intersect with social and cultural niche factors to produce different risks for different people based on their gender, geography and socio-economic status. Social relations of gender and power can determine access and control over household resources and shape lay perceptions of illness, drugs and health (9). AMR is linked to human, animal and environmental health, and in pastoralist populations, it both shapes and is shaped by cultural practices and social organisation related to livestock rearing, agriculture and the associated gender roles (10).

Undoubtedly, approaches to understanding these complex drivers must go beyond one discipline and integrate a wide range of expertise and understandings. They must be based on a One-health approach, recognising that humans, animals, plants and the environment are interconnected and indivisible, at the global, regional and local levels, and from all sectors, stakeholders and institutions (2, 11).

The One Health approach is gaining momentum as a strategic framework in AMR research, however, the lack of attention to some of the deep drivers of AMR, particularly in community settings impacts the effectiveness of this approach. For example, One Health has not considered how gender inequities in access to resources, including medical resources such as drugs, shape exposure to antimicrobials, particularly in informal health systems in low-resource settings (12, 13). As Garnier and colleagues [(13): 7] rightly argue, One Health and its proponents “are still failing to consider the critical importance of adopting and implementing a gender-sensitive One Health approach, not only to respect a human-rights dimension but also to bring transformative change in finding new sustainable pathways to the environmental, health and climate crisis that we are facing”.

### 2.1 Gender and risk of AMR

Gender is critical in understanding the risks of AMR across communities. Gender plays an important role in shaping roles and interactions between people, livestock, the environment and health systems, which has implications for health outcomes (14).

In livestock-keeping communities across Africa and Southeast Asia, research has shown how infections linked to animal-keeping have gendered risks of exposure both to infections (such as zoonoses) and to antimicrobials used in treating livestock, through meat and milk consumption (10, 14). Among East African pastoralists including in Tanzania, women and girls are more likely to be exposed to these risks due to their gender roles in animal health and welfare (4, 14, 15). Men and women in these communities have varying degrees of exposure to antibiotics, during administering medicines to livestock including ingesting aerosols, in contexts where appropriate PPE is scarce (16). Women are likely to face greater risks of exposure through their roles in gathering and preparing food, with contacts including dirty food preparation surfaces and source contamination during food gathering. In Zimbabwe for instance, as is the case in many stallholder livelihoods, women and girls spend more time in forests than men and boys, foraging for food and medicine, and where they are at a higher risk of exposure to vector-borne zoonoses, and are routinely treated with unprescribed antibiotics (17). Gender drivers of AMR must therefore be explored and incorporated into interventions to meet the differential health needs in communities.

### 2.2 Gendered access and use of antimicrobials

There is widespread evidence of overuse of antibiotics worldwide, which may exacerbate the spread of antimicrobial resistance. A recent report by the WHO shows a surge in antibiotics prescriptions during COVID-19, during which some 75% of patients were treated with antibiotics “just in case” they helped, even though there was no evidence of bacterial co-infections. Antibiotic prescriptions decreased in the Americas and Europe over time between 2020 and 2022, but it increased in Africa and the Eastern Mediterranean over the same period (18).

Excessive and inappropriate use of antibiotics is a major driver of AMR particularly in low-resource settings where regulation of supply for pharmaceuticals is limited. For example, antibiotic consumption increased globally by 65% from 2000 to 2015, with most of the rise in LMICs, and it is projected to triple between 2015 and 2030 if no action is taken (19). People can misuse antibiotics by not completing the prescribed course of antibiotics, using the incorrect dosage or being prescribed antibiotics unnecessarily. These practices can allow more resistant bacteria to thrive, multiply and potentially lead to the development of superbugs that are difficult to treat with existing antibiotics (2, 20).

However, few studies have focussed on how gender determines the availability, access, and use or misuse of antimicrobials. Limited evidence from Bangladesh and Nepal shows that more men than women purchase antibiotics without a prescription, although women are more likely to be prescribed antibiotics by clinicians than men, and women are more likely to use a prescription than men (20, 21).

Sex differences in disease epidemiology are much more researched because of long-standing awareness of the role of biology in disease distribution [see (22, 23)]. For example, women's experiences associated with pregnancy, abortion and childbirth may put them at increased risk of antibiotic resistance, especially when combined with unsafe and unhygienic healthcare, inadequate water and sanitation, insufficient and/or unaffordable antimicrobials, and inadequate knowledge about appropriate medicine use (24). Meanwhile, more men smoke than women, with research showing that smoking affects vulnerability to infectious respiratory diseases such as influenza and tuberculosis, and therefore men are at a higher risk of these infections compared to women (22). As men and women's bodies age, they interact with infections and treatments differently across their life course. Women may require antibiotics more during childbearing years while men may be prescribed these more often in older age, for example, shifting vulnerabilities and exposure in unique ways (22, 23).

## 3 Study context

### 3.1 Study context: Tanzania

#### 3.1.2 AMR-one health application in Tanzania

AMR is a particular concern in Tanzania, which has the 175th highest age-standardised mortality rate per 100,000 population associated with AMR across 204 locations (IHM, n.d). The number of AMR deaths is higher than from respiratory infections and tuberculosis, maternal and neonatal disorders, neoplasms, HIV/aids and sexually transmitted infections, and neglected tropical diseases and malaria.

A review of 10 studies of antimicrobial susceptibility testing (AST), carried out between 2012 and 2019 on patient samples at major zonal and referral hospitals in Tanzania, showed that the most frequently identified microorganisms among in-patients included Staphylococcus aureus, Klebsiella pneumoniae Escherichia coli and Pseudomonas aeruginosa, and resistance of these organisms to common antibiotics including ampicillin and amoxicillin/clavulanate ranged between 30% and 100% (ibid). These micro-organisms are a major cause of hospital-acquired infections such as surgical site infections, urinary tract infections, bloodstream infections and pneumonia, with the multi-drug resistant (MDR) strains much more prevalent in intensive care units in LMICs, due to inadequate hygiene and infection control measures (25). Several studies have found that women are at higher risk of infection from these strains than men (26–28).

Tanzania developed a National Action Plan for AMR (2017–2022) to tackle AMR using a One Health AMR coordination mechanism. Consequently, a One Health AMR Surveillance Framework was developed to guide the establishment of AMR surveillance systems in the human, animal and environmental health sectors (29, 30).

Presently, a new 5-year National Action Plan against AMR (NAP-AMR) 2023–2028 is in place and it incorporates six strategic objectives: Strengthen coordination, collaboration and governance for NAP on AMR implementation; Create awareness and understanding of antimicrobial resistance through effective information, education and communication; Strengthen the knowledge and evidence base through surveillance and research; Reduce the incidence of infection through effective sanitation, hygiene, infection prevention, and on-farm biosecurity; Optimize the use of antimicrobial medicines in human and animal health; and develop the economic case for sustainable investment that takes into account the needs to increase investment in new medicines, diagnostic tools, vaccines and other interventions (ibid).

Despite these strides, challenges remain around commitment from the various ministries, implementing institutions in both the public and private sectors and other AMR stakeholders (NAP-AMR, 2023–2028). While the Action Plan is grounded in a whole system approach extending from the individual level to the macrosystem with clear multi-sectoral and inter-disciplinary involvement using the One Health Approach, gender is not incorporated into the plan nor is there a focal point for gender considerations. The challenge of frequent access to and unregulated use of antimicrobials in Tanzania requires the incorporation of livelihood and socio-cultural beliefs that determine exposure to AMR risks.

### 3.2 Study site: Naiti, northern Tanzania

**Figure 1:**
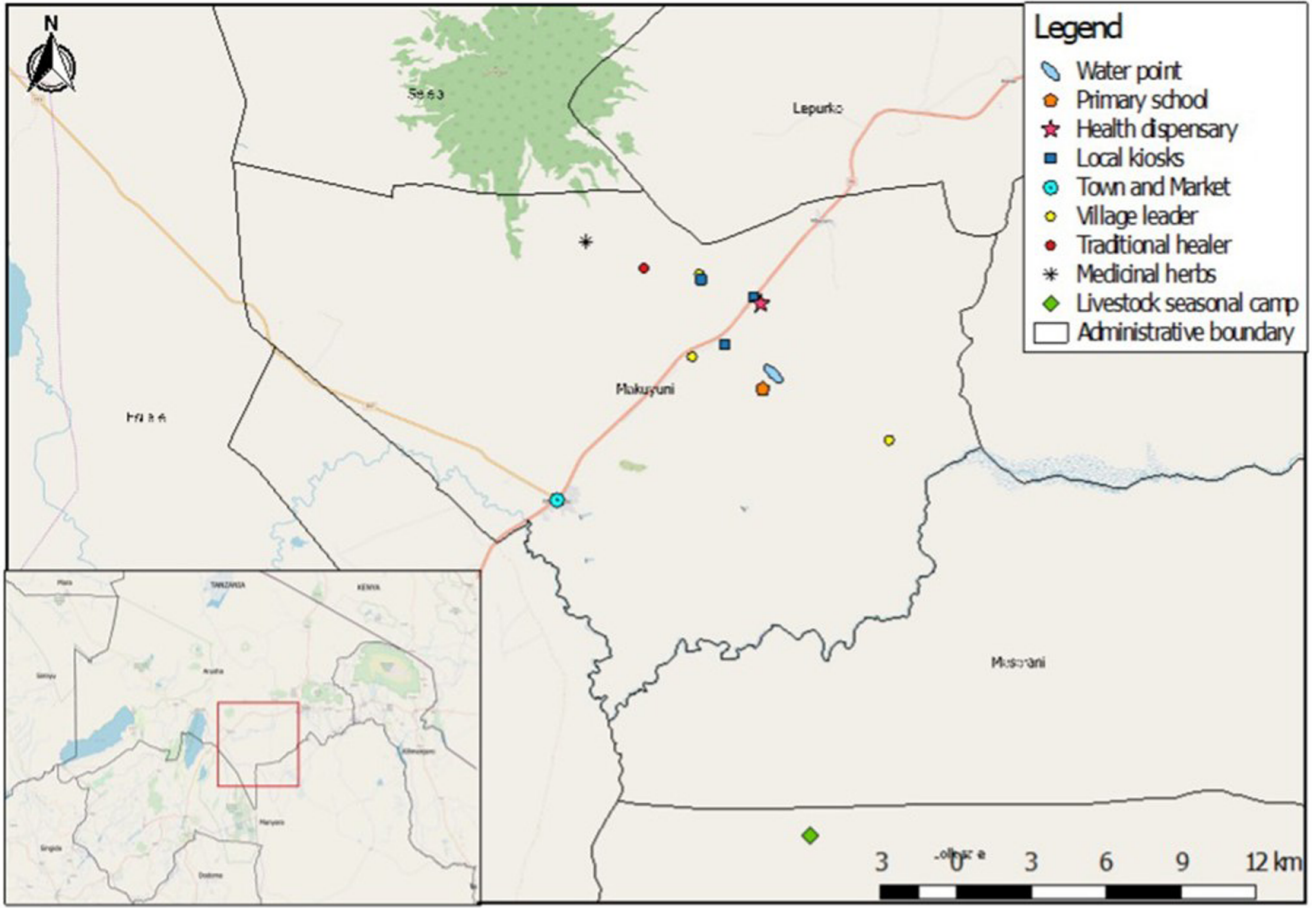
Map of the Naiti area and its surrounding villages.

**Figure 2:**
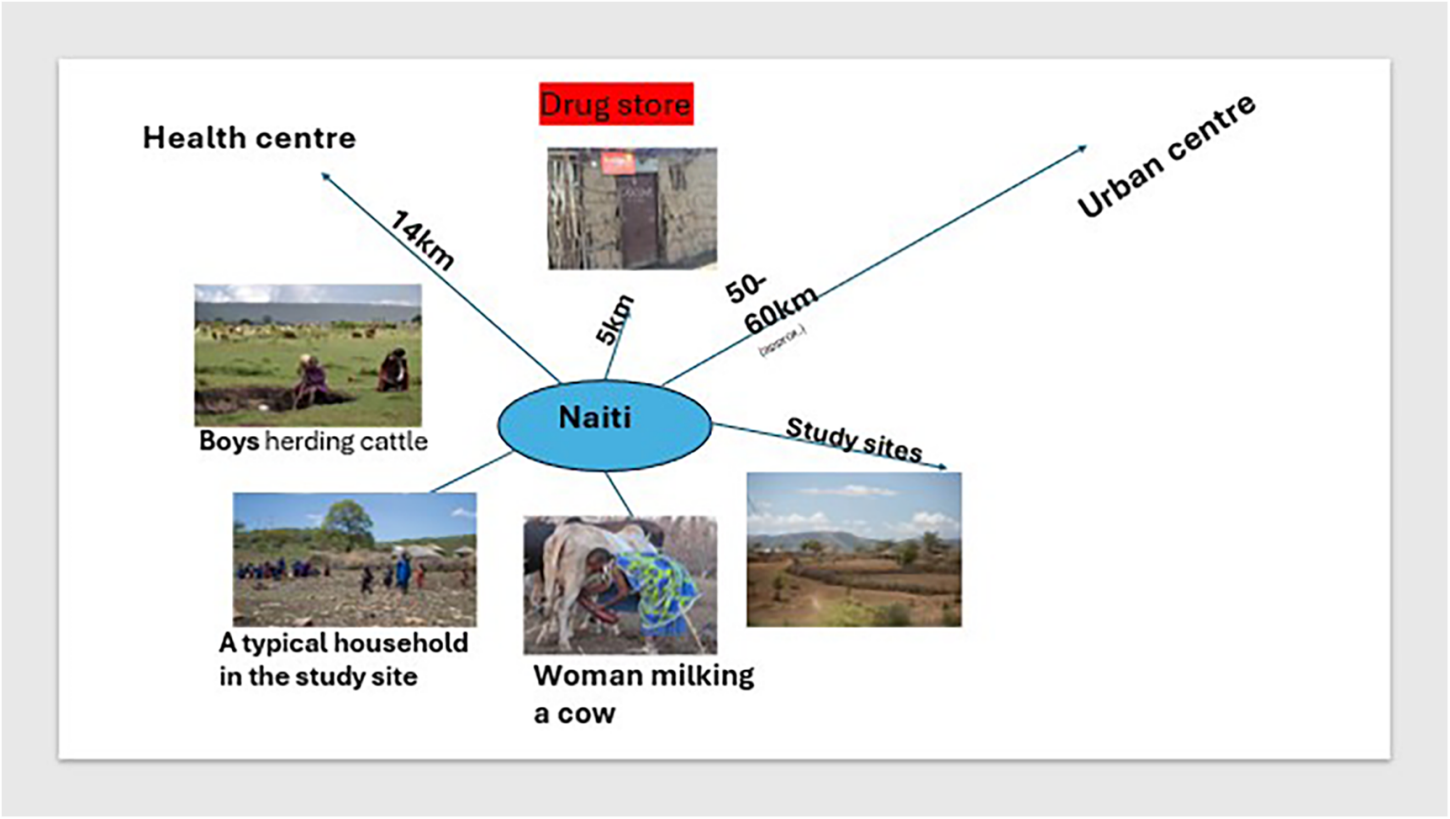
Naiti landscape: animal/human/environment/health systems interactions.

This study took place in four pastoralist villages in Naiti, northern Tanzania. Naiti and its surrounding villages lie within the Maasai Steppe (31). It is an area of vast savannah plains located along the Arusha-Manyara Road in Makuyuni ward, Monduli district, northern Tanzania (see Figure 1). Its inhabitants can be best described as agro-pastoralists, that is, in addition to livestock production, they are also involved in crop cultivation (10). Most people here own small plots of land where they grow food crops and raise their livestock and are not wholly transhumant.^1^ As Figure 2 shows, women typically tend to milk cows and are involved in milking and processing dairy products, feeding and managing their health, while men and boys herd cattle, sheep and goats, and travel vast distances looking for pasture and water for the animals.

Pastoral and agro-pastoral communities live in some of the most remote and hard-to-reach areas in Tanzania, characterised by poor public health infrastructure that results in limited healthcare options for many families. At the time of this study, there was only one basic health dispensary Naiti village, which was established in 2014 (see Figure 2, inset). The clinic was staffed with one clinical officer^2^ who was responsible for a population of about 3,000 people spread across a vast area, with a poor road network making it difficult for many to access the dispensary. The health clinic in the area was located approximately 14 km^2^ from most Naiti inhabitants and was operating under severe constraints, lacking medicines, diagnostic equipment, staffing, electricity and water. Consequently, for most of their health problems, families rely on a range of informal providers who fill the gap of unmet demand in healthcare. These include traditional healers and drug peddlers, who flourish but are not well-regulated by the government. A small, makeshift shop (in Figure 2) sold medicines and doubled up as a drugstore and a grocery store. It was the first point of interaction between patients and the informal health system. People walked typically 5 km one way to access the “shop” and would only ever consider walking much further to the health facility (14 km each way) if they were seriously ill and at the advice of the “drug” storekeeper.

### 3.3 Methods

The overall methodological approach for this research was mixed methods, with ethnography at its core. The methods used included survey techniques, semi-structured and open-ended interviews (key informant and in-depth), and ethnographic observations in various local settings, including of clinical diagnoses at a local health dispensary and of drugs frequently purchased at the local shop (pictured in Figure 2) in Naiti. The decision to combine qualitative and quantitative methods was not merely to provide a comparison of findings, but as Pope and Mays (33) also noted, an attempt to explore complex issues by employing methodological diversity.

The study took place between 2016 and 2017 as part of a PhD field research which focussed on lay approaches to the treatment of febrile illness in pastoralist communities in northern Tanzania. The study focussed on people's interpretation of febrile illness, treatment-seeking behaviour and interaction with both formal and informal health systems. Following ethical approvals and after obtaining consent from participants, data collection strategies involved unstructured participant observation of participants in homes and sites of healthcare provision. A total of 379 adult participants from 200 households (see Table 1) were purposively sampled from a possible 659 households across four villages. Most respondents (94%) were semi-literate, with only five participants reporting having completed primary education.^3^

**Table 1:** Data collection schedule and no of participants.

200 households (HHs): 2 respondents from 179 HHs, 1 respondent in each of remaining HHs				
Total = 379 participants	Male	Female	Total	Timeline
HH survey (all participants)	168	211	379	Oct. 2016–Jan. 2017
In depth interviews	24	23	47	March 2017
Key informant interviews	17	9	26	May 2017
Focus group discussions	50	50	100	May–June 2017
Observation of clinical diagnoses	20	10	30	Oct. 2016–June 2017

The purposive sampling technique was appropriate because as Bernard (34) advises, and in the case of Naiti where the population is highly homogenous, this approach helped to attain a sample that best represented the features that would answer the research questions. Variables considered included: distance to the health facility, age, sex, gender, education, marital status and socio-economic status.

A structured survey questionnaire was developed by the author in collaboration with local research assistants following an iterative process, and it was based on a rigorous literature review. It was translated into local languages by local researchers familiar with local dialects. The questionnaire included five modules:

1) livelihoods (how families derive a living, including food, shelter, water, medicines),
2) human-animal interaction (who, where, when human-animal interaction occurs),
3) framings of risk for zoonotic disease (what is risky and what is not, how different people perceive risks of animal illness, and how risk is managed locally),
4) experiences with febrile illness (including lay interpretation of symptoms and labelling for febrile illness),
5) healthcare-seeking behaviour (where people seek treatment and how decisions are arrived at).

All the 379 participants responded to the survey questionnaire, after completing consent forms. Consent was obtained either in writing (for those able to) or verbally followed by thumbprints (for those unable to sign). Verbal consent was obtained in the presence of at least one other adult member of the household. Questionnaires were administered to an adult member of the household, either the household head his wife, or another adult responsible for health-related decision-making in the household.

Survey responses were clustered into themes and a preliminary analysis informed the development of interview guides for qualitative research, where key themes about gender dynamics and health-seeking behaviour were probed further.

A total of 46 in-depth interviews with 23 women and 24 men and 26 key informant interviews with 17 men and nine women were conducted. The interview guides contained both structured and semi-structured questions. Structured questions were based on “Yes” or “No” answers by asking a single item question, such as: Have you (in the last 2 weeks) or are currently taking any medicines? Which ones, can you show me? Semi-structured questions explored broader attitudes towards antibiotic administration (history of antibiotic taking within the previous 2 weeks, awareness of dosage, where these are purchased, by whom and for what purposes), and knowledge of which diseases are or are not treatable with antibiotics.

The responses were corroborated following a presentation to participants during focus group discussions (FGDs). FGDs involved 50 men and 50 women, with an average group size of five members per session. The Focus group discussions were constituted as disaggregated by sex and by gender, as was the analysis of the FGDs.

During these discussions, participants were presented with summarised findings from the survey questionnaire, translated into the local language by field assistants, and presented on flip charts. A ranking exercise was then used to rank different types and names of drugs mentioned as commonly used by survey respondents and in interviews. These included allopathic drugs such as painkillers, antibiotics and syrups for children, to traditional medicine obtained from the forest such as leaves and bark of trees. These group discussions also provided an opportunity to identify issues that participants converged around or that resulted in divergent views or opinions, and these were then used to refine further interview guides.

Facility-based observations (at the health centre, see Figure 2) were followed by interviews of the patients in their homes regarding diagnosis, prescription whether this was obtained and from where the drugs were obtained. Participant observation at the local drugstore involved documenting the exchanges between the seller and the buyer and whether drugs were purchased based on a prescription.

The local storekeeper was a young man in his early to mid-30s; he had no formal education. However, as a pastoralist, he was experienced in local illness therapies and seemed to know exactly what his patients would want to buy. In our interview, he shared that he mostly stocked paracetamols and amoxicillin as they were the most popular drugs. The medicines were removed from their original packaging that contained dosage information and were instead repackaged in smaller doses (2–6 capsules in each bag) and priced accordingly.

Participant observation also occurred at the health clinic, involving observing diagnosis and prescription by the clinician, who consented in writing to these observations. Only patients and or their accompanying families who consented were observed. Consent was either verbal or in thumbprint. The clinician was also a young man in his mid-30s and was assisted by a female nurse. He arrived at diagnoses based on symptoms and the patient's illness history alone as he had no diagnostic tools or electricity at the facility. Where he thought symptoms needed further examining, like those requiring x-rays, CT scans or blood tests, he referred patients to the district hospital in Arusha, approximately 60 km away (see Figure 2). Most patients with these referrals could not travel to Arusha due to lack of transport, and they ended up self-treating with antibiotics purchased from the local shop.

At the clinic, information gathered included the patient's demographic characteristics, given diagnosis and type of prescription if any. A follow-up interview with the patient and/or their family gathered information on the illness prognosis, treatments sought and whether a clinician's prescription was followed through. These opportunities offered me access to what Gubrium and Holstein [(35): 38] explained as “sufficient proximate experience of the everyday circumstances in which people learn and tell their stories,”

## 4 Data analysis

In-depth interviews and FGD data were analysed thematically using NVivo coding software and theme generation. A codebook was developed based on the identified stratifiers guided by the gender analysis approach (23), interview guides, and other emerging themes from interviews and FGDs. The initial codebook was refined throughout the analysis process following several iterations through the various data profiles. This process generated the key findings for this study.

The next section on findings will focus only on the fifth module: healthcare-seeking behaviour (where people seek treatment mostly, which drugs are bought, by who and how decisions are arrived at, to understand gender dynamics of access to and use of antimicrobials and the associated risk of AMR.

## 5 Findings

**Figure 3:**
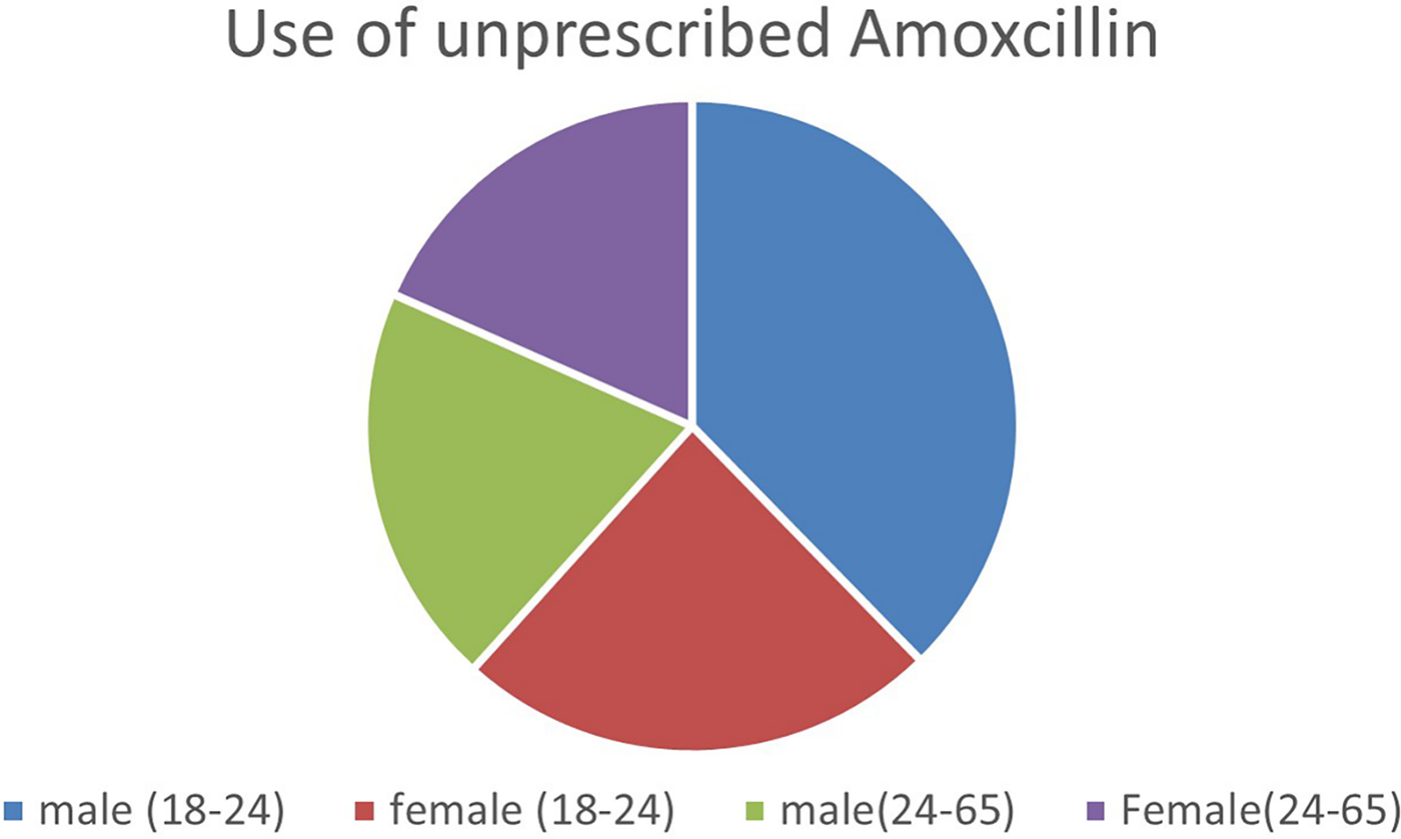
Main findings.

As Figure 3 above illustrates, young men aged between 18 and 24 who are mostly unmarried boy herders purchased and ingested unprescribed amoxicillin the most, while young women of the same age, who were all married to an older male used amoxicillin slightly less than their male counterparts but their numbers were still Age difference in antibiotic use among older participants aged 24–65 did not vary significantly observations corroborated this finding as younger men (18–24) were the most frequent customers who purchased amoxicillin from the local shop, whereas older participants were more likely to visit the healthcare dispensary when they deemed an illness too severe to treat at home. These instances involved children's health and the elderly.

### 5.1 Health-seeking behaviour in plural health systems

This study found that in remote pastoralist settings, health-seeking is complex and dynamic and is rooted in collective and interactive processes between lay people and local health systems, playing out in ways that are complex and that go beyond a single episode of illness or an individual family Equally, decisions on where to seek healthcare, what healthcare and for whom are also influenced by these complex familiar negotiations, and by multiple interpretations and perceptions of illness both at home and in sites where health-seeking occurs. Younger men and women may be more exposed to antibiotics than older men, shaped both by their exposure to the risk of illness (herders may come into contact with pathogens in bushes where they spent most of their time with livestock, while younger women are more likely than older women to forage for food, firewood in similar places).

In the study sites, informal healthcare providers are relied upon for the bulk of healthcare needs for many families. While the survey and ethnographic study focussed on treatment-seeking behaviour for fever of unknown causes, most families used the same routes of treatment (purchasing unprescribed antibiotics) for all illnesses that were interpreted as “ordinary” illnesses.^4^ Undoubtedly, febrile illness was just but one of many ailments with multiple and complex socio-economic, environmental and other drivers that are common in low-resource settings. However, febrile illness, presenting with symptoms of fever or “hotness” as was locally identified, seemed, in this study, to be quite common, and treatment-seeking followed familiar regimens. Families self-administered antibiotic drugs (obtained from friends and families or bought from the local shop.

In other more severe cases of febrile illness and ones where people believed the patient could fare worse off if and after administering antibiotics at home, the caregiver, often the male head of the household consulted the informal providers who were somewhat trusted to have basic medical knowledge. These included unlicensed, and untrained drug sellers (often referred to as “bwana dawa” or *medicineman* or “dokta” or *doctor*) who stocked the drugs, again, mainly antibiotics because there was a huge market for them in these communities. The sellers advertised themselves as able to offer diagnostic and therapeutic medical advice to patients and their families; including, which medicines to take for an illness. While some people did trust them, there was also a fair amount of people, particularly older people, who seemed to question such advice.

**Figure 4:**
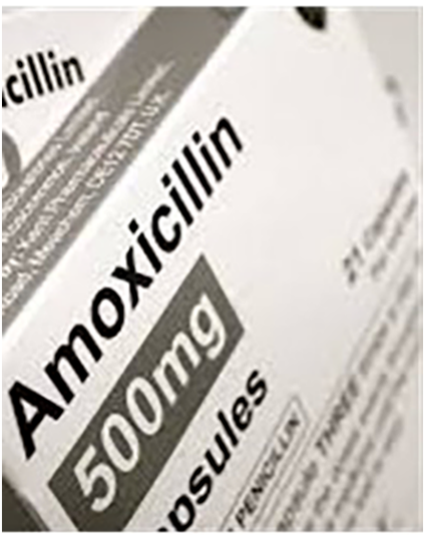
A packet of amoxicillin at the shop in Naiti.

**Figure 5:**
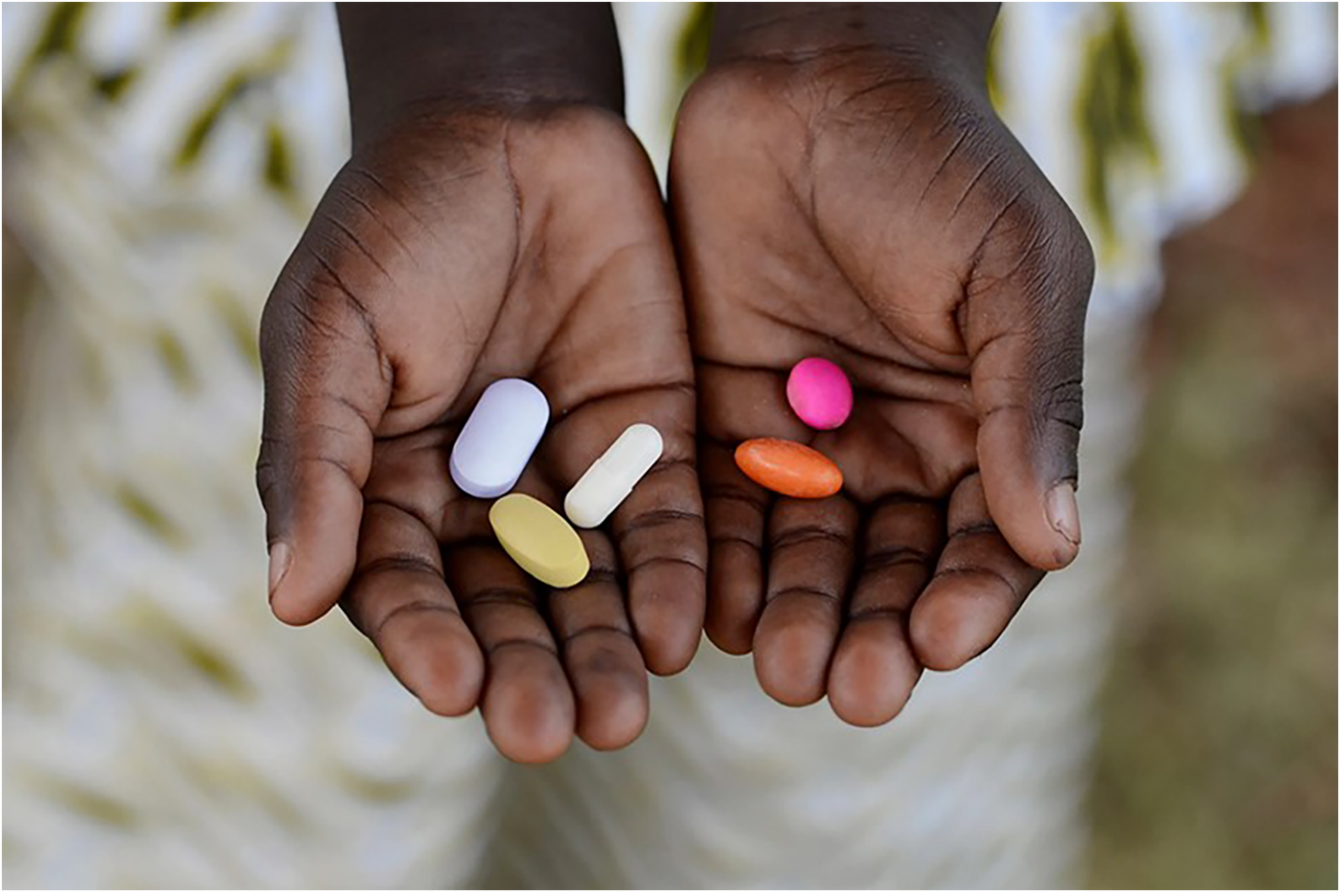
A participant holding amoxicillin and paracetamol drugs.

Unlicensed drug sellers purchased and stockpiled both allopathic and herbal medicines in bulk from retailers operating in trading centres or pharmacies in urban centres such as Arusha, a much bigger town centre with traders from all around the region. The drug shop owner from where observations in this study took place operated from a basic “shop” in the village where he also sold general household merchandise, alcohol and tobacco. My interview with the drugstore owner, a young male from a nearby pastoralist family, indicated that his medical stocks were mainly of antibiotics (amoxicillin) (see Figures 4, 5) and paracetamols because these were very popular drugs and “they sell quickly because everybody wants them”.^5^ He, like other drug sellers inside or outside the villages, operated outside any regulatory framework, selling drugs that were unlabelled, in clear plastic bags, often in small quantities and with no dosage information. I however confirmed that the storekeeper did buy amoxicillin drugs in their original packaging but then repackaged these into smaller doses because as he told me, “No one can afford the whole packet (dose) at once”.

**Figure 6:**
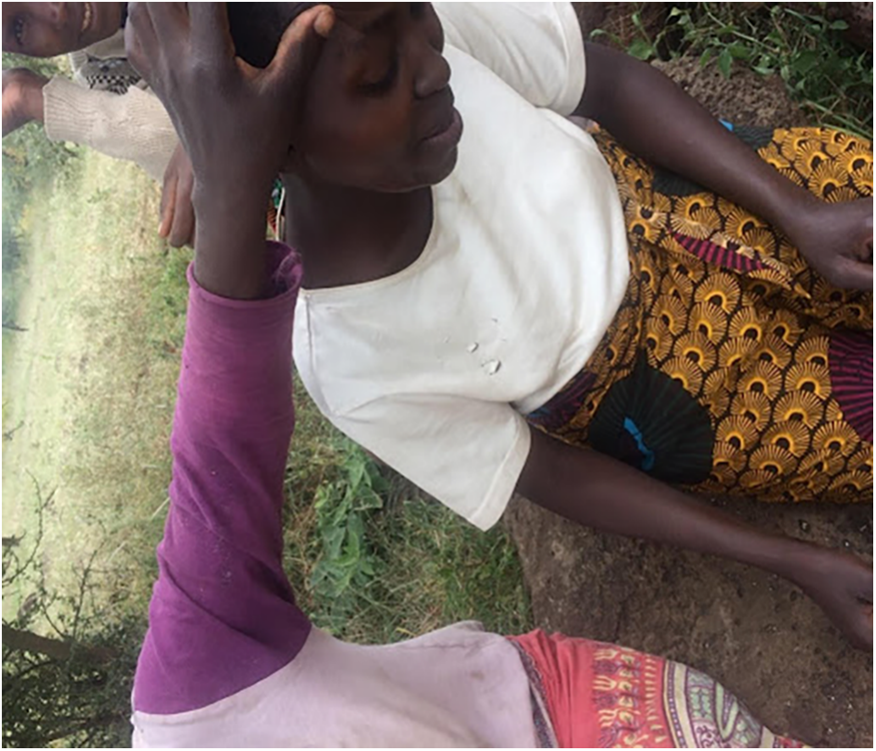
An older woman examining a younger woman's illness symptoms. Credits: author.

### 5.2 Knowledge and awareness of AMR

In their National Action Plan (2023–2028), the government of Tanzania recognises the large gap in community on awareness, risk communication and education on AMR. Because AMR is often an invisible problem with multiple drivers and affects multiple systems, including people, livestock and the environment, it can be complicated to explain to communities. Even clinical staff, operating in medically under-resourced settings can face difficulties in approaching a correct diagnosis and prescription. In Monduli, where this study took place, the existence of but one health facility with a clinical officer and a nurse and serving a population of over 3,000 people, who are scattered across vast distances meant that many families relied on informal health providers for their healthcare needs.

As one would expect, few participants in this study, including the shop owner were aware of correct use of antibiotics. They thought the drugs were stronger than paracetamol and patients and their families were not aware of information regarding dosage and duration as people often purchased drugs in small quantities and administered to patients only until the patient seemed better, usually 3 days after starting the course.

Young men and women (18–24 year olds) who were also more likely to use antibiotics without prescription, seemed to be least aware of the correct use of the drugs. Some responses on the question: Do you know what amoxicillin is used for, included:

Amoxicillin treats pain quickly. If I feel joint pains after a long day with cattle in the bushes, I take one capsule of amoxicillin before going to sleep, when I wake up the following morning, I take another one. I normally feel better after two days. It is a very good drug^6^

I swallow amoxicillin to help with chills and fever. This is not for malaria but for other painful fevers, when I have throat pain or pain in my chest then I know I need amoxicillin to treat it.^7^

I know amoxicillin can treat swelling in the legs. My legs swell often, maybe because of my age. But I don't like amoxicillin because it doesn't not make you better forever, you have to keep swallowing it every few days. I prefer herbs from the forest because they can heal me completely.^8^

This study finds that age and gender factors are significant determinants of unprescribed antibiotic use. Younger people viewed antibiotics as “quick fixes” to their health problems and did not regard them as a threat when misused, older people, on the other hand, were more cautious about the actual benefits of taking antibiotics, with oe describing them as unable to “heal completely” perhaps about the fact that if improperly used, antibiotics would not be effective in disease management.

Meanwhile, 46% (*n* = 187) of customers indicated using the same antibiotics to treat both human and animal sickness. These interviewees shared that,

My two cows have the same sickness as me; they are coughing too, and they have diarrhoea. I think I got the illness from them [suggesting a zoonotic connection]. I have bought some amoxicillin I will mix with the water for the cows, and I will take some of the tablets as well.^9^

Animals are just like us, if they fall sick, you treat them, and they get better. What is wrong with using the same medicine? Their bodies work the same way as ours^10^

The local *doctor* [drugstore owner] advised me to take two tablets of amoxicillin every four hours. He said I might have a chest infection because I was coughing a lot, and my body was hot.^11^

These examples illustrate that human and animal health beliefs and understandings are embedded and reflected in complex ways of everyday life in pastoralist communities. In Naiti, a significant majority of participants used the same modalities to interpret symptoms and assign labels to febrile illness in both people and livestock; the sense of animals and humans as sharing a lifeworld is deeply embedded and is reflected in narratives of despair, with illness and therapies to treat these co-constructed from each other.

Where febrile illness was deemed too complicated to treat at home, usually after several days of home treatment, and where the patient's condition remained the same or worsened, the family would take the patient to the clinic (a wife would be accompanied by her husband or a senior male relative, a man would go on his own and children would be taken by their mothers). Key informant interviews with the clinician showed that the decision to attend the clinic is usually the last one and can often mean that the illness has progressed to the point where the patient's chances of recovery are threatened. The clinician in the health centre in the villages commented that,

Normally when a patient comes here, they have already tried everything else, antimalarials, herbal treatments, prayer etc., so they come when it is late, and they tell me they have malaria. When I test and find there is no malaria, they ask if it is typhoid, or increasingly, urinary tract infections. But I cannot tell them for sure because I cannot confirm these diseases, so I give them general advice. When they leave here they go straight to the drug store and buy antibiotics without knowing what they are treating.^12^

Patients, particularly women, expect to be diagnosed with malaria at the clinic and to be prescribed antimalarial drugs when they perceive their febrile symptoms to constitute “severe/serious fever”. Men generally did not visit the clinic when they thought they had malaria (they self-treated with herbal therapies mixed with fresh milk,) but went to the clinic if they interpreted their severe febrile symptoms as pneumonia or tuberculosis, and they expected to be prescribed antibiotics. When clinicians cannot reach a diagnosis (due to a lack of diagnostic facilities) or where antibiotics are not prescribed (in case of a viral or other infection), patients try to obtain antibiotics themselves outside of formal healthcare settings. The clinician disclosed that,

When I tell a patient that they do not have malaria or pneumonia, they do not believe it and they want antimalarials or antibiotic drugs. If I say no, you cannot have these, they will still buy the drugs from the sellers and ingest them.^13^

Families seemed to stock antibiotics in their homes in anticipation of illness, and they frequently shared these among sick family and friends without consultation with a health professional. A patient told me in an interview:

Sometimes we borrow amoxicillin from friends and family if we have similar sickness. There is usually someone in our family who has the medicine at any given point because we know the sickness [febrile illness] is common…^14^

Perhaps these examples also suggest that people in Naiti are aware of the limitations of formal health systems (limited diagnostic and human resource capacity) and are therefore less likely to rely on clinical diagnosis that is based on symptoms alone. Because of improvements in the diagnosis of malaria in Tanzania, other studies have noted Malaria to be a more readily acceptable diagnosis than other illnesses and missing malaria in the diagnosis is deemed “inexcusable” (34). A non-malarial fever was therefore seen as only treatable with antibiotics, mainly penicillin and amoxicillin, both of which were locally believed to be an effective treatment for severe fever.

#### 5.2.1 Lay diagnosis and referrals as predictors of antibiotic use

In Naiti, lay referrals are consulted to identify and label illness, particularly if symptoms are of “unusual fever”, a common description for febrile illness that does not respond to antibiotics within three days of commencing self-treatment.

Lay referrals in Naiti are comprised of older and experienced members of the family such as older men and senior women (over 65 years of age) who may also be traditional although they do not always have to be. Patients approach these people, who either collaborate in labelling illness or, where they are unfamiliar with symptoms, advise patients to visit a trained expert, sometimes a medical doctor and sometimes a local healer. See Figure 6, showing a young woman being examined by older women who are part of her lay referral network. In turning to lay networks for referral during illness, patients are seeking relatable illness experiences, as some expressed to me:

When we are ill, we go to my grandfather to tell us what the illness is. He knows many illnesses and their symptoms because he has lived for a long time and has seen many of the illnesses.^15^

We have a group of people we go to when there is a health problem. If it is an ordinary fever, you do not need to consult them, but if you have a serious [severe] fever, they can tell you which one it is because they are experienced and knowledgeable about illness and treatment.^16^

All of them [senior members in the family] have experienced this or another illness in their lifetime and they know if you have a similar illness or not. If they tell me to take my child to the hospital, I trust them, if they say, this is ok, you can deal with it at home, I also listen to the advice because I trust them.^17^

Lay referrals complicate decisions on access to therapy because they may and often advise patients to take antibiotics to “kill the germs and herbal remedies to ‘cleanse the blood’. A patient who described her lay referral circle as ‘better than the hospitals’” said in an interview:

My grandmother has been helping with my and my children's sickness. First, she asks us to take penicillin because it kills the germs that cause sickness, but we must also drink the herbs after the sickness is cured so that it can help flush the remnants of the antibiotics from the blood and make the body clean.^18^

Arguably, lay people understand the dangers of taking unprescribed antibiotics but seem powerless to do something about it, in a context where access to health services is limited by a host of intersecting challenges (poverty, illiteracy, and political marginalization to name but a few).

### 5.3 Gendered decision-making and health-seeking behaviour

The final theme that emerges from this study, and which is closely related to lay referrals is decision-making for seeking treatment within the household. Established pastoralists’ social structures place all decision-making in the hands of men and/or senior women (including mothers-in-law, first wives in polygamous unions and female healers). For almost all treatment options available to the household, it is husbands (or male relatives if husbands are and/or senior women) who make decisions regarding which treatment to pursue. This group is also often part of the local lay referral systems. Wives are involved in deciding treatment options for themselves or their children when treatment is free, as in herbal therapies, or when relying upon already-stocked pharmaceuticals, either in the household or obtained from neighbours. Women forage for herbal remedies from the forest and mix them with milk or meat soup and blood, before either ingesting them or applying them topically to the affected part of the body. A female febrile patient explained that:

When I am ill, I start with what I can personally afford. I have to go to the forest because there is no fee for herbs. But with drugs, my husband can buy penicillin or ask a family member to share, depending on what illness I have.^19^

Gender intersects with other determinants of health-seeking behaviour in these communities, such as health-system barriers (inadequate diagnostic facilities, staff shortages, language barriers) and socio-economic barriers (high transportation costs, poverty, illiteracy), to shape health outcomes through the differential exposure to intermediary determinants of health, and ultimately to differentiated risk exposure to antimicrobial resistance (6, 35). For febrile patients, particularly when they are girls or younger women, it is their gender that influences their access to antibiotics as they are less likely to go against medical decisions made by lay referrals on their behalf. Husbands and senior women may “diagnose” and “prescribe” antibiotics based on their widely accepted wisdom and experience with illness, even when this wisdom is contrary to what the patient himself or herself may believe to be true. In one case, a young woman who believed her febrile illness to have been a result of drinking dirty water and having “stomach fever” and was against taking penicillin tablets because she did not believe it to be an effective treatment, was nevertheless “forced” to take antibiotic tablets by her mother-in-law. She explained that,

My symptoms are not body fever, but stomach upset because I drank unclean water from the well yesterday while I was at the river cleaning the laundry. I think once I flash out the dirt from my stomach with herbs, I will feel better. I do not need the hospital medicine [antibiotics]. However, my mother-in-law believes my symptoms are similar to what she experienced two weeks ago, and she got better after taking the drugs, so she has insisted that I take the drugs too, and she has given me two tablets.^20^

Gender not only plays out in how access to drugs including antibiotics is negotiated but also in the ways that men and women, young and old, perceive their illness and symptoms, which in turn determines health-seeking behaviours. For example, a young male herder who was visibly ill did not consider visiting a doctor despite having taken amoxicillin tablets for 3 days. He explained to me that:

A man should not always go to a doctor when ill. It makes you weak. I do not need a doctor because he will tell me to take more tablets. I know what to take and I have taken amoxicillin for 3 days but because I see no change in my health, I will stop taking them and try herbal medicine^21^

Here, the gendered norms and expectations of when to visit a doctor when ill and the perception of strength and weakness influenced the herder's behaviour. The idea that the herder believed that a doctor would prescribe antibiotics meant that he did not trust the health system to be better at arriving at a different diagnosis than his own and he therefore went ahead and took amoxicillin without a prescription, disregarding dosage and stopping taking them three days into it. This has implications both for early detection of illness and on exposure to AMR in these and similar contexts.

## 6 Discussion

### 6.1 Gendered access to antimicrobials

As this study demonstrates, gender plays a key role in access to antibiotics. Gender relations of power are a major social determinant of health (35). As a power relation, gender influences decisions around health-seeking, from when and where to seek healthcare to what healthcare is sought. Barasa and Virhia's (6) research in Tanzania also showed that husbands and male guardians in pastoralist families often make decisions regarding where women and girls can access healthcare. In many cases, the researchers found men purchased antibiotics without a prescription and brought them home to sick wives suffering from undiagnosed febrile illness. In Jones et al.'s (20) study, similar findings are reported in Nepal, where, while health posts are described as accessible to women, only men are mentioned in interview transcripts as accessing private health facilities and pharmacists. A female participant in this study notes that.

*…the women are a bit shy in nature. There are some women that do not step out of their house at all. Some women do not even get on the public vehicles. So, the women are not able to go to the hospitals that are far away and get checked up there* [(20): pg.6]*.*

Additionally, drug sellers are often exclusively male (as is the case in the present study, and in both Jones et al.'s and Barasa and Virhia's studies in Nepal and Tanzania respectively).

Research increasingly shows that in LMICs, unlicenced drug sellers, both peddlers and shop-based, are an important source of access to medicines (36, 37). This is because they offer affordable and accessible healthcare in settings where a vast majority of the population is unreached by formal healthcare providers.

In pastoralist settings which are often in remote areas informal drug sellers are almost the only way people interact with the health system. In formal healthcare settings, gender and age shape prescribing practices for antibiotics. Smith and colleagues (38) explored gender in antibiotic prescribing in the literature involving nine countries, and they found that women received more antibiotics than men in all age groups except in over 75 categories, with women aged 16–54 receiving 36%–40% more antibiotics than men of the same age. These differences are both a function of sex (that women experience certain diseases such as UTI, more frequently than men, and they require antibiotics to treat them) and also of gender. Schröder and colleagues (39) showed that women are far more exposed to AMR than men and indicated that they are 27% more likely to receive an antibiotic prescription in their lifetime compared to men. Socio-cultural and biological factors compound women's vulnerability to antibiotic prescription. Biological factors such as childbirth, menstruation, abortion, and socio-cultural barriers to access to health information and restricted health choices compound women's vulnerability to ABU and by extension, to AMR (23).

In addition, some studies show that men and women communicate differently with healthcare professionals, and women may readily accept antibiotic prescriptions from male healthcare providers either because they are too shy to question a male provider, as in the case of Jones et al.'s (20) study or, male providers may have biases that influence antibiotic prescription during consultations with women (38, 39) or both of these scenarios. Ultimately, it is important to recognise gender as a crucial determinant of AMR-driving behaviours in community settings and it is therefore essential to incorporate gender in holistic approaches such as One Health in AMR research.

Alongside gender roles that dictate that women and girls are the caretakers of sick animals, which exposes them to risks of zoonotic microbes, people frequently used the same antibiotics to treat animal and human diseases, at times simultaneously, particularly where symptoms were perceived to be similar. Interviews and FGDs with household members revealed deep-seated beliefs that animals and their owners share the same disease risks and that treatment of these need not be different. Vector-borne diseases such as malaria, common in pastoralist areas in Tanzania, especially during rainy seasons, were also believed to affect animals. As such, families stocked up medicines during the drier months in readiness for wet-season disease outbreaks, during which the same medicines would be used to treat symptoms in both people and their livestock.

The threat of AMR in these communities is exemplified by the lack of withdrawal from animal products while the said animals undergo treatment using antimicrobials. For Example, families consumed milk from sick cows undergoing treatment, and if the animal died, the carcass was consumed as it was believed to be safe. Again, due to gendered household food distribution practices that involve women and children nursing sick milk cows, this group is likely to be at an elevated risk of exposure to antimicrobials, firstly, through proximity to sick animals and secondly, through consumption of animal products from sick animals undergoing treatment.

## 7 Conclusions: opportunities for using gender and one health in AMR research

Researchers applying a One Health approach to AMR must consider how gender is entangled with global health challenges not just in LMICs but in developed country contexts as well. Arguably, the drivers of AMR are complex and dynamic, and different drivers interact with each other in mutable ways that make it difficult to address using a single approach. Allel et al. (40) highlighted the most common transmission routes for drug-resistant organisms including human-to-human transmission, exposure to contaminated medical equipment, medical waste, food and environments, as well as through animal-human interactions. These transmission routes are gendered, and there is a need to look at how gender influences drivers of AMR and how the consequences of this are distributed across individuals and groups particularly those living in low-resource settings (41). Yet, One Health approaches have traditionally lacked focus on gender and intersectional drivers of infections in community settings. Garnier et al. (13) point out researchers need to “acknowledge that One Health will only be able to fulfil its vital purpose if some of the deep drivers of the inter-connected crises are addressed”.

The findings in this paper mirror findings from a growing body of research that demonstrates the influence of gender on AMR. Jones and colleagues' (20) study in Nepal shows gender as a major determinant of antibiotic use. This Study shows how gender inequalities influence access to health facilities and impact patterns of prescribing behaviour by clinicians in Nepal, where not only are clinicians likely to be male but they are also likely to prescribe antibiotics based on symptoms alone, and more often to their female patients than male ones. In community settings, gender inequalities are also manifested through gender norms, roles and resource distribution, including medical resources that influence health-seeking behaviours.

While research into gender and AMR is starting to take shape (41), there is little in the literature on how holistic approaches such as One Health can utilise gender as a cross-cutting driver of infections and the consequences of this for the burden of AMR in LMICs community settings.

As LMICs continue to grapple with the effects of changes in the environment, including climate change impacts on food security, health and livelihoods, people working in healthcare, agriculture or food production will become increasingly more vulnerable to AMR, as they are exposed to resistant bacteria in the animals and humans they interact with. Antimicrobial resistance is also likely to interact with the growing threat of climate change and its impacts, particularly among at-risk groups including pastoralists and smallholder farmers across sub-Saharan Africa and East Asia, who are least prepared to adapt to the changing climate (42). Without urgent interventions that consider gender and other social determinants of health, AMR will exacerbate health inequalities that are already at alarming rates across the globe. As van der Heijden and colleagues (45:6) put it, “poverty feeds the problem of antibiotic resistance”, creating a vicious cycle that undermines the development achievements of the past decades.

Finally, as a policy entry point, therefore, enhanced collaboration between the animal and human health sectors is important, however, policymakers must also pay attention to informal health actors, including lay health systems and co-opt these into regulatory frameworks for access to antibiotics. Secondly, One Health approaches can be strengthened by incorporating gender and other social factors that determine health-seeking behaviour, to better understand and target interventions to those at the greatest risk of antibiotic use, including women and girls, and young male herders. As women and girls are likely to be deprioritised for treatment when resources are scarce, they are more likely to stay sick for longer and use various unprescribed medicines, both of which can exacerbate their exposure to and susceptibility to developing a DRO and AMR.

## Data Availability

The raw data supporting the conclusions of this article will be made available by the authors, without undue reservation.

## Footnotes

^1^The residents practice some form of transhumance; they usually split their herd, leaving the “milk” herd permanently at the homestead, and sending the rest on short transhumance.

^2^A clinical officer is a person with at least 3 years of post-schooling basic and applied medical training. As these officers are trained to only provide basic health services, the law requires that they are supervised by a more senior health care practitioner, where they are deployed (in the main at the district and divisional levels), this is not always the case in reality (32). For example, in the study villages, these people acted as physicians for all kinds of health problems.

^3^Primary education in Tanzania is 7 years, with the average age of starting school being 6 years old.

^4^Ordinary was perceived as illness that did not immediately constitute a threat to life.

^5^Interview with drug seller, Naiti. 11 July 2017.

^6^Interview with young male herder, Naiti, 2 July 2017.

^7^Young female participant, Naiti 25 July 2017.

^8^Key ifnromant interview with male elder, Naiti 14 August, 2017.

^9^Interview with a woman in Naiti, 10 May 2017.

^10^Focus group discussion with young male herders, Naiti, 4 May 2017.

^11^Interview with a male participant, Naiti, 18 May 2017.

^12^Interview with the clinician in Naiti dispensary, 12 July 2017.

^13^ibid.

^14^Interview with a female patient in her home, following self-treatment with antibiotics.

^15^Interview with female patient in Naiti.

^16^Discussant in an FGD.

^17^Ibid.

^18^Interview with a female patient in Naiti on 19 July 2017.

^19^Interview, Naiti, 29 June 2017.

^20^Interview with a young woman with symptoms involving stomach pains, Naiti, June 2017.

^21^Participant observation, and interview with young male herder, May 14 2017.
